# Differential Bacterial Surface Display of Peptides by the Transmembrane Domain of OmpA

**DOI:** 10.1371/journal.pone.0006739

**Published:** 2009-08-25

**Authors:** Gertjan S. Verhoeven, Svetlana Alexeeva, Marileen Dogterom, Tanneke den Blaauwen

**Affiliations:** 1 Molecular Cytology, Swammerdam Institute for Life Sciences, Faculty of Science, University of Amsterdam, Amsterdam, the Netherlands; 2 Department of Biomolecular Systems, FOM Institute for Atomic and Molecular Physics (AMOLF), Amsterdam, the Netherlands; Instituto Butantan, Brazil

## Abstract

Peptide libraries or antigenic determinants can be displayed on the surface of bacteria through insertion in a suitable outer membrane scaffold protein. Here, we inserted the well-known antibody epitopes 3xFLAG and 2xmyc in exterior loops of the transmembrane (TM) domain of OmpA. Although these highly charged epitopes were successfully displayed on the cell surface, their levels were 10-fold reduced due to degradation. We verified that the degradation was not caused by the absence of the C-terminal domain of OmpA. In contrast, a peptide that was only moderately charged (SA-1) appeared to be stably incorporated in the outer membrane at normal protein levels. Together, these results suggest that the display efficiency is sensitive to the charge of the inserted epitopes. In addition, the high-level expression of OmpA variants with surface-displayed epitopes adversely affected growth in a strain dependent, transient manner. In a MC4100 derived strain growth was affected, whereas in MC1061 derived strains growth was unaffected. Finally, results obtained using a gel-shift assay to monitor β-barrel folding *in vivo* show that the insertion of small epitopes can change the heat modifiability of the OmpA TM domain from ‘aberrant’ to normal, and predict that some β-barrels will not display any significant heat-modifiability at all.

## Introduction

Bacterial surface display is widely used to screen peptide libraries for e.g. epitope mapping and selection of high affinity binders [1]. As differences in display efficiency among different clones could cause unwanted biases during the selection process, knowledge of the capabilities, as well as the limitations of the particular display scaffold used, is considered to be of importance [2].

One such scaffold is the OmpA protein in *Escherichia coli*. OmpA is an integral outer membrane protein (OMP) embedded in the bacterial outer membrane (OM) as a β-barrel. It contains four surface exposed loops in which peptides can be inserted that subsequently are displayed on the cell surface [3]. Several reports exist in which the OmpA protein has been used as a bacterial surface display system, for applications such as peptide library screening [4], [5] use in a novel selection strategy [6], use as live vaccines [7] or to sequestrate cadmium for bioremediation [8].

The full-length, processed OmpA protein (325 residues) consists of two domains, a N-terminal transmembrane (TM) domain of 170 residues, connected via a short 19-residue Ala-Pro rich hinge region to a C-terminal periplasmic domain of 136 residues [9]. The periplasmic domain plays a structural role by tethering the OM to the peptidoglycan cell wall layer. However, genetically truncated OmpA-171 consisting of only the TM domain assembles into the outer membrane as efficiently as the full-length protein [10]. For a comprehensive review on OmpA structure and function see [11].

Our goal was to display an epitope on the bacterial cell surface that could be recognized by commercially available antibodies and used as a handle in biophysical force experiments (to be published elsewhere). OmpA was chosen because it is very abundant (typically about 10^5^ copies/cell (Koebnik et al. 2000)) and widely studied [12], [13]. For our biophysical application, adverse effects of peptide-insertion on protein levels or OM insertion were unwanted. Because epitope-sized insertions in loop 2 or 4 of OmpA have been shown to be tolerated without negative effects [3], this served as an additional reason to choose OmpA. Thus, we have inserted the epitope tags 3xFLAG and 2xmyc into loop 2 and 3 of the transmembrane domain of OmpA (here also referred to as OmpA-177), and studied their stability and outer membrane incorporation *in vivo*. As the cell wall anchoring by the periplasmic domain was unwanted in these experiments, the TM domain of OmpA was initially used instead of the full-length protein.

We show that these engineered OmpA TM domain variants can be incorporated into the OM and that the epitopes are successfully displayed on the cell surface. To our surprise however both are present at roughly 10-fold reduced levels compared to the β-barrel without epitope due to degradation. Since to our knowledge, all *in vivo* loop insertions to date have been made in full-length OmpA, we investigated a possible stabilizing role of the periplasmic domain. Our data demonstrate that the periplasmic domain did not stabilize the 3xFLAG insertion variants. These results suggest an incompatibility of 3xFLAG and 2xmyc tags with the OM biogenesis pathway of OmpA. In the end, we created an OmpA insertion variant that met our requirements by truncating a full length OmpA containing an SA-1 peptide identified in a search for streptavidin binding peptides [5]. The results obtained with this variant demonstrate that it is possible to display a peptide in the OmpA β-barrel without negative effects on protein levels or OM insertion. Together, these results suggest that the display efficiency is sensitive to the charge of the inserted epitope, as both 2xmyc (20-mer) and 3xFLAG (22-mer) are highly charged (50% and 70% charged residues, respectively) whereas the SA-1 peptide (15-mer) is moderately charged, containing only 2 charged residues.

In addition, we report that the high-level expression of epitope-containing OmpA constructs had a strong, transient, negative effect on the cellular growth rate that was strain dependent. In a MC4100 (a standard *E. coli* laboratory strain) derivative, growth rate was transiently impaired, whereas in strain MC1061 as well as a ΔOmpA MC1061 derivative no detrimental effects were observed. This suggests that when over-expressing outer membrane proteins, MC1061 is a strain of choice.

To test for outer membrane incorporation, we make use of OmpA's so-called heat modifiability [14]. In its folded form, OmpA migrates to a different position in SDS-PAGE compared to its (heat denatured) unfolded form [15]. Whereas for full length OmpA, the folded form migrates faster through the gel, for the transmembrane domain of OmpA it is the other way around [10]. This was termed ‘aberrant heat modifiability’ [10]. We show that small epitope insertions in the transmembrane domain restore normal heat modifiability, and this observation predicts that some β-barrels might not exhibit any heat modifiability at all.

## Materials and Methods

### Bacterial strains and growth conditions

*E. coli* strains (Table 1) were grown at 37°C in TY medium containing 1% Bacto trypton, 0.5% Bacto yeast extract, 0.5% NaCl and 3 mM NaOH. Expression of the constructs was induced by adding up to 1 mM IPTG or 0.02% L-arabinose, depending on the plasmid vector. Antibiotics were ampicillin (100 µg/ml) or Chloramphenicol (25 µg/ml). LMC500 (MC4100 *lysA*) was made chemically competent using the calcium chloride method. MC1061 and its derivative MC1061 ΔOmpA were transformed using electroporation.

**Table 1:** Strains and plasmids used in this study.

Strains	Genotype	Reference
LMC500 (MC4100 *lysA*)	F^−^, *araD139*, Δ*(argF-lac)*U169, *deoC1*, *flbB5301*, *ptsF25*, *rbsR*, *relA1*, *rpslL150*, *lysA1*	[20]
MC1061	F^−^, *araD139*, Δ(*ara-leu*)7696, Δ*lac*X74, *galE*, *galK*, *hsdR2* (r_k−_ m_k+_), *mcrA0*, *mcrB1*, *rpsL*, *spoT1, relA1*	[22], [38]
MC1061 ΔOmpA	MC1061 ΔOmpA	[5]
DH5α	F^−^, *endA1*, *hsdR17*(r_k−_ m_k+_), *supE44*, *thi-1*, *recA1*, *gyrA*, *relA1*, Δ(*lacZYA-argF*)U169, *deoR*, Φ80 *lacZ*ΔM15	Lab collection
DH5α-Z1	DH5α LacI_q_^+^ TetR^+^	[39]
Plasmids	Proteins expressed	Reference
pMD005	pTHV037 OmpA-177	This work
pGV1	pTHV037 OmpA-177 2xmyc in Loop 2	This work
pGV2	pTHV037 OmpA-177 3xFLAG in Loop 2	This work
pGV3	pTHV037 OmpA-177 2xmyc in Loop 3	This work
pGV4	pTHV037 OmpA-177 3xFLAG in Loop 3	This work
pGI9	pTHV037 OmpA-LEDPPAEF	This work
pGI6	pTHV037 OmpA-LEDPPAEF containing 3xFLAG in Loop 3	This work
pB33OmpA14-SA1	pBAD33 OmpA SA-1 in Loop 1	[5]
pGV28	pTHV037 OmpA-177-SS SA-1 in Loop 1	This work
pGV32	pTHV037 OmpA-LEDPPAEF 3xFLAG in Loop 2	This work
pGV33	pTHV037 OmpA-LEDPPAEF SA-1 in Loop 1 OmpA-177	This work
pTHV037	pTRC99A with a weakened IPTG inducible promoter	[16]

### Constructs

All constructs (Table 1) were cloned into a pTrc99A vector (Pharmacia Biotech, USA), a pBR322 derivative plasmid, of which the *trc* promoter was modified with a down mutation to reduce expression levels [16]. All DNA manipulation, analysis and bacterial transformations were performed according to standard protocols (Sambrook et al., 1989). All PCR fragments were sequenced, either at Baseclear (Leiden) or at the AMC DNA sequencing facility (Amsterdam Medical Centre). Primers were ordered from MWG or Biolegio, and Advantage DNA polymerase (Clontech) or *pfuTurbo* DNA polymerase (Stratagene) was used for the PCR reactions. The cloning steps performed to obtain the plasmids are described in the Materials and Methods S1.

### Preparation of cell lysates

Fresh overnight cultures grown at 37°C were diluted 1000x into 50–100 ml fresh TY medium and cultured at 37°C. Growth was monitored by measurement of the optical density at 600 nm with a spectrophotometer (Perkin-Ellmers). IPTG was added at around an OD600 of 0.1, and when the cells reached an OD600 of 1.0, they were transferred to a 50 ml Falcon tube and put on ice. The cells were then collected by centrifugation for 15 min at 4000 rpm in a tabletop centrifuge at 4°C (Eppendorf). The supernatant was carefully removed, and the cells were resuspended in ice-cold sonication buffer (10 mM Tris-HCl buffer, pH 7.9, supplemented with 1 mM EDTA and 1 tablet of Roche Protease Inhibitor Cocktail), at a concentration corresponding to an OD600 of 250. This cell suspension was transferred to a 2 ml Eppendorf tube, and sonicated on ice with a tip sonicator (Branson) in 4–5 10-second bursts with 10 second cooling in between each burst. Debris and intact cells were pelleted in a 4°C cooled centrifuge at 2700 x g for 2 min. The supernatant was transferred to a 1.5 ml Eppendorf tube and frozen at −20°C as total cell lysate.

### Fractionation of cell lysates

After thawing, the cell lysate was diluted to 4 ml (corresponding to an OD600 of 12.5), and 100 µl of this was saved as “total cell lysate”. The samples were pelleted at 45000 rpm (corresponding to 200.000 x g) for 45 min in an ultracentrifuge (Beckman-Coulter). After centrifugation, 500 µl was saved as “supernatant”. The membrane pellet was resuspended in 100 µl sonication buffer and frozen at −20°C.

### SDS-PAGE and Western blotting

For SDS-PAGE, samples were mixed with sample buffer (end concentration: 62.5 mM Tris pH 6.8, 2% SDS, 10% glycerol, 2% 2-mercaptoethanol) and either heated to 99°C for 5 min or heated to 50°C for 15 min and electrophoresed on 15% polyacrylamide slabs. Anti-FLAG and anti-myc monoclonal antibodies used for the immunoblots were obtained from Sigma and Roche, respectively. The polyclonal anti-OmpA antibody was a kind gift from A. Driessen (University of Groningen, Netherlands). The bands were detected using the ECL+ chemiluminescence kit (Amersham) and scanning with a STORM 860 fluorescence imager. Densitometry was performed using ImageJ (http://rsb.info.nih.gov/ij/). The mean pixel value of a rectangular region was calculated close but outside a band of interest to calculate the mean background pixel value. The same selection rectangle was positioned to include the band of interest, and again a mean pixel value is calculated. Subtraction then gives a band intensity value. All band comparisons were performed using the same selection rectangle.

### Fluorescent labeling of fixed cells

Cells were fixed in 2.8% formaldehyde (FA) and 0.04% glutaraldehyde (GA) in growth medium for 15 min at room temperature, then washed and resuspended in PBS (140 mM NaCl, 27 mM KCl, 10 mM Na_2_HPO_4_·2H_2_O, 2 mM KH_2_PO_4_ pH 7.2). Cell concentration was adjusted to an OD600 of 0.6 and samples were incubated in 75 µl PBS containing 30 mg/ml BSA to block non-specific sites on the cell surface for 30 min at 37°C. Then antibodies were added, either anti-FLAG (M2, Sigma) or anti-myc (9E10, Roche) at an end concentration of 20 µg/ml, and samples were incubated at 37°C for 30 min. The cells were washed 3 times with 2 volumes of PBS containing 30 mg/ml BSA, and then incubated in 1 volume with Donkey-anti-Mouse-Cy3 conjugate (Jackson ImmunoResearch) at 10 µg/ml end concentration for 30 min at 37°C, washed 3 times with 2 volumes PBS and imaged.

### Fluorescent labeling of living cells

Cells were put on ice, and an amount of cells equivalent to 1 ml OD600 of 0.3 (around 2·10^8^ cells) was taken for labeling. Cells were collected in all cases by centrifugation at 20.000 x g for 5 min at 4°C. The pellet was resuspended in 75 µl PBS at room temperature (RT) with 0.1% BSA. The cells are left at RT for 10 min to block aspecific sites on the cell surface. Then either biotinylated anti-FLAG (Sigma) was added (50 µg/ml) (FLAG constructs), or streptavidin-Alexa 488 (Molecular Probes) was added directly (40 µg/ml) (SA-1 constructs). Cells were incubated at RT for 30 min. The cells were spun down and washed twice with 0.5 ml PBS, and resuspended in 150 µl PBS. For the cells labeled with biotinylated FLAG, streptavidin-Alexa 546 (Molecular Probes) was added (5 µg/ml), and samples were incubated for 30 min at RT. Then, PBS (0.85 ml) was added and the cells were pelleted. After a second wash with 0.5 ml PBS, the cells were fixed in 1 ml PBS with 2.8% formaldehyde and 0.042% glutaraldehyde, washed in 1 volume of PBS and resuspended in 0.1 volume PBS. The cells were either imaged directly or stored at 4°C over night before imaging.

### Fluorescence Microscopy

Cells were immobilized on 1% agarose in water slabs-coated object glasses as described by [17] and photographed with a CoolSnap *fx* (Photometrics) CCD camera mounted on an Olympus BX-60 fluorescence microscope through a UPLANFl 100x/1.3 oil objective (Japan). Images were taken using the public domain program Object-Image2.19 by Norbert Vischer (University of Amsterdam, http://simon.bio.uva.nl/object-image.html), which is based on NIH Image by Wayne Rasband. In all experiments the cells were first photographed in the phase contrast mode. Then a fluorescence image was taken using either a green excitation/red emission (U-MNG, ex. 530–550 nm), or a blue excitation/green emission filter cube (U-MNB or EGFP, ex. 470–490 nm).

## Results

### Design of loop insertions

**Figure 1:**
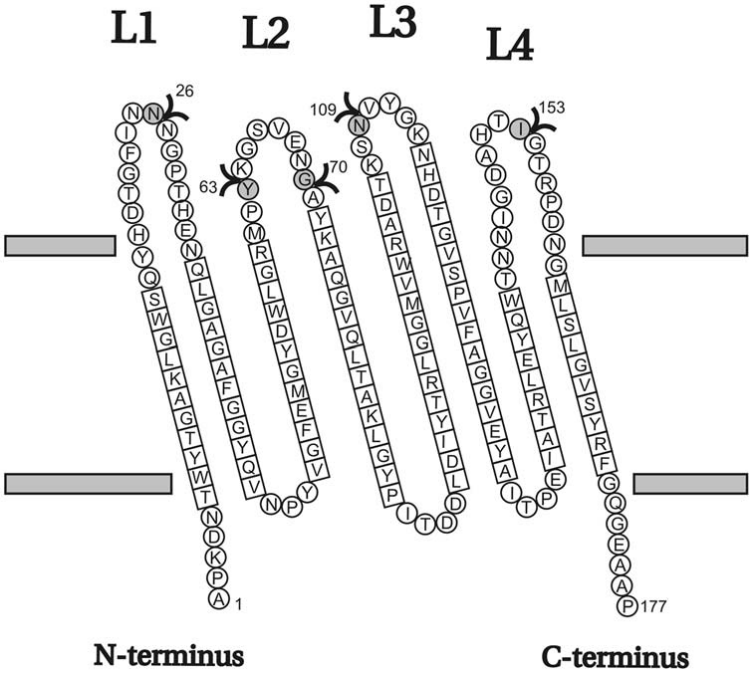
Topology model of the TM domain of OmpA (OmpA-177) (adapted from [40]). Black arrowheads indicate positions where peptides have been inserted: after N26 [5] and this study, Y63 [18], G70 [3] and this study, N109 this study, and I153 [3], [41]. Residues present in β-strands are indicated with squares. Other residues are presented as circles.

A topology model of the transmembrane domain of OmpA is shown in Figure 1. For locations in loop 2 and loop 4 (after Y63, G70 and I153 respectively), it has been shown that small (up to 21 residue) peptides can be inserted without any reduction in protein levels [3], [18] and membrane incorporation (after G70 and I153, [3]). For loop 2, reported inserted peptides are listed in Table 2. Initially, we used only the OmpA TM domain, but later also the periplasmic domain was added. 3xFLAG and 2xmyc peptides were chosen as epitope tags (Table 2). We will refer to them as FLAG and myc from now on. High-affinity monoclonal antibodies are commercially available for these epitopes. SWISS-Model [19] was used to predict OmpA folding after peptide insertion. First, a continuous model was generated of the first 176 residues, based on the published crystal structures of OmpA-171 (Figure S1A). Then, models were generated of loop insertions after different residues in the protein, and the resulting (static) loop conformations were examined for their propensity to extend away from the membrane normal axis. Models were generated of insertions in loop 2, 3 and 4. Finally, loop 3 was chosen, since the computer-generated model of an inserted FLAG peptide after N109 predicted the largest distance away from the surface (Figure S1C).

**Table 2:** OmpA peptide insertions in loop 2 as reported in literature as well as this study.

Name	Target	Loop	AA	Sequence	−/+ Charge	Reference
8 AA	pronase	L2	8	NWLGRMPY	0/1	[18]
21 AA	proteinase K	L2	21	AGMQAYRIRA RYPGILFSRPA	0/4	[3]
3xFLAG	mAb M2	L2/3	22	DYKDHDG-DYKDHDI-DYKDDDDK	−11/4	This study
2xmyc	mAb 9E10	L2/3	20	EQKLISEEDL EQKLISEEDL	−8/2	This study
SA-1	Streptavidin	L1	15	RLEICQNVCYYLGTL	−1/1	[5]

To be able to compare the performance of our constructs with results reported in literature, we also constructed loop 2 insertions with the FLAG and myc epitopes. For loop 2, insertions after G65, G70, Y72 and Q75 were modeled. In the end G70 was chosen, because (a) as mentioned, it was shown that at this location, a 21-residue peptide could be inserted without any negative effects on membrane insertion [3], and (b) modeling with SWISS-Model of these four locations predicted that at G70, the loop would be extended away from the surface more than at the other three positions in loop 2 (Figure S1B).

During the course of this work, a loop 1 peptide insertion (SA-1 tag) in full-length OmpA was described in the literature [5]. The position of this insertion is also indicated in Figure 1 (after N26). As mentioned in the introduction, the SA-1 peptide tag (listed in Table 2) binds streptavidin directly with high affinity. Since this peptide was neutrally charged and could be conveniently labeled with fluorescent streptavidin, we decided to compare this loop insertion variant with our constructs. Therefore, it was cloned into our expression vector, both as a truncated OmpA consisting only of the TM domain and as a full-length protein, and assayed in the same way as the FLAG/myc insertions.

### Growth of cells expressing OmpA-177 loop insertion proteins

**Figure 2:**
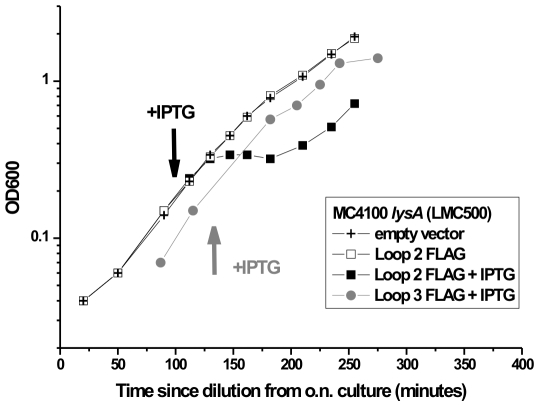
Growth curves of cells expressing OmpA TM domain variants: effect of IPTG induction. Shown are growth curves of OmpA-177 variants with and without induction with IPTG. Note that FLAG inserted in Loop 2 causes a growth delay upon induction whereas FLAG inserted in Loop 3 does not. Similar behavior was observed for the myc tag (data not shown, see also Table 3). Cultures grown over night at 37°C were diluted 500 times in fresh TY medium. At an OD_600_ of 0.2, IPTG was added to a final concentration of 0.3 mM when indicated. Crosses represent control cells harboring the empty plasmid (pTHV037). Squares represent cells that express OmpA-177 with a FLAG insertion in loop 2 (Open: without IPTG, Filled: with IPTG). Circles represent cells that express OmpA-177 with a FLAG insertion in loop 3 and IPTG added.

The constructs were tested for expression in LMC500 (MC4100 *lysA*), a well characterized laboratory strain [20], [21], MC1061 ΔOmpA [5], an OmpA knockout strain, and in its parental strain MC1061 [22]. To test to what extent the proteins could be expressed without affecting the growth rate, the growth was followed by measuring the optical density before and after addition of the inducer IPTG. Growth curves from a typical experiment are shown in Figure 2. In this experiment, cells carrying an empty vector were compared with cells expressing an OmpA-177 protein with a FLAG insertion either in loop 2 or loop 3. Without IPTG induction, all growth curves appear identical. Approximately 15 minutes after addition of IPTG however, the OmpA-177 protein with a FLAG insertion in loop 2 shows a lag phase where growth stops for approximately 30 min, after which growth continues normally. In contrast, for the growth curve of the OmpA-177 variant containing a FLAG insertion in loop 3, no lag phase is observed after induction with IPTG. Instead, the growth curve is similar to the growth curve of uninduced cells.

**Table 3:** Growth and molecular mass as detected on immunoblot after induction with 0.3 mM IPTG of LMC500 expressing OmpA proteins with loop insertions.

Construct	Peptide	Location	Growth after induction	Mass on PAGE/blot (kDa)
MD005	none	NA	++	19
GV2	3xFLAG	loop 2	−−	25
GV4	3xFLAG	loop 3	+	25 and 27
GV1	2xmyc	loop 2	−−	25
GV3	2xmyc	loop 3	+	25 and 27
GV28	SA-1	loop 1	–	21

*“Growth after induction” indicates the presence and extent of a lag phase after adding inducer (++ no lag phase, −− strong lag phase)*.

The induction experiment was performed for all the OmpA TM domain variants and the results are listed in Table 3. It can be concluded that in strain LMC500, induction of the loop 2 (FLAG/myc), and to a lesser extent, the loop 1 (SA-1), but not the loop 3 (FLAG/myc) insertions caused a lag phase of 30–60 min after which growth continued normally. Surprisingly, this lag phase was absent both in MC1061 and in MC1061ΔOmpA. Induction with 0.3 mM IPTG of the loop 2 (FLAG), or loop 1 (SA-1) in these strains had no effect on the growth rate at all. Because in LMC500 and MC1061, similar protein levels were detected on immunoblots, it appears that strain MC1061 and its derivative MC1061ΔOmpA are more able to cope with the perturbation caused by the induction (see also discussion).

### Expression of OmpA-177 loop insertion proteins

Samples, harvested at the end of the induction experiment described above were analyzed by SDS-PAGE and immunoblotting. For the FLAG and myc constructs, a blot is shown in Figure S2. Based on the immunoblots it was clear that all four variants (either FLAG or myc in Loop 2 or 3) were expressed, no degradation bands were observed, and all ran at a similar height that was retarded with respect to their calculated molecular weight (approx. 3–4 kDa). This was also observed for full-length constructs that carried a FLAG insertion (see below). The reduced mobility on gel was attributed to the high amount of negative charge in the FLAG and myc peptides. Unexpectedly, unprocessed (precursor) proOmpA-177 was also detected in uninduced samples as well as in induced samples expressing either FLAG or myc in loop 3 (see also Figure S3). The difference in protein levels between induced and uninduced samples was a factor 2–5 fold (all protein levels were determined from immunoblot by densitometry using ImageJ, data not shown). Without induction, for FLAG as well as myc, about 25% of the loop 3 constructs detected were not processed.

**Figure 3:**
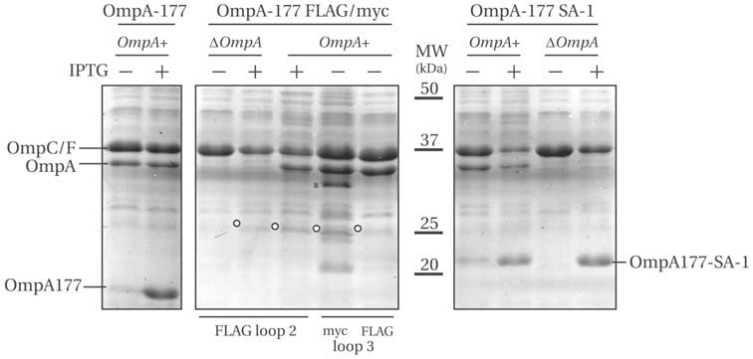
OmpA-177 TM domain variants containing FLAG or myc, but not SA-1 are present at reduced levels. Shown is a Coomassie stained 15% SDS-PAGE containing membrane fractions. Fractions corresponding to 1 ml OD_600_ of 0.625 cells were loaded. IPTG was either absent (−), or added during exponential growth at an end concentration of 0.3 mM (+). Membrane fractions were isolated both from LMC500 (*OmpA+*) and MC1061 ΔOmpA. Open circles indicate the expected position of the FLAG and myc variants as determined from immunoblotting (e.g. Fig. S3). The asterisk indicates an unknown band that did not react with anti-myc on immunoblot. The positions of the OmpC/OmpF band, as well as the endogenous OmpA band are indicated.

Induced OmpA-177 without loop insertion was directly visible on a Coomassie Brilliant Blue (Coomassie) stained SDS-PAGE (see Figure 3) and could be detected on immunoblot using a polyclonal antibody against OmpA, but the band was very weak compared to endogenous (full-length) OmpA (data not shown). From this, we conclude that this polyclonal antibody primarily recognizes the periplasmic domain of OmpA. Also, an attempt was made to detect the streptavidin binding peptide SA-1 construct on blot using a Streptavidin-HRP conjugate, but only the endogenous cytoplasmic biotinylated biotin carrier protein BCCP was detected (data not shown). Apparently, streptavidin does not bind sufficiently strong to the denatured conformation of SA-1.

To be able to directly compare the amounts of protein in the membrane, membrane fractions were isolated of cells expressing the various OmpA TM domain variants (Figure 3). Induction of both OmpA-177 and OmpA-177 SA-1 constructs resulted in strong bands at roughly their expected height (calculated MW of OmpA-177 is 19.3 kDa and of OmpA-177 SA-1 is 21.1 kDa). Surprisingly, no bands could be identified that corresponded to the FLAG or myc constructs.

To determine how much of the expressed construct is present in the envelope fractions put on gel, we compared soluble (S) to membrane (M) fractions. Although not visible on a Coomassie stained gel, immunoblots of the fractions showed that the FLAG and myc constructs were present, and fractionated predominantly to the membrane fraction (Figure S3). We conclude that these constructs are present in the cell at greatly reduced amounts (less than 10% as judged from the Coomassie stained gel) compared to wild type OmpA-177 or the OmpA-177-SA1. This could either be due to a reduced synthesis rate (not expected for FLAG and myc tags), due an increased degradation rate (see also below) or both.

### Role of the periplasmic domain

To our knowledge, all OmpA loop insertions reported in the literature have been made in full-length OmpA. To establish whether the periplasmic domain might play a role in preventing a reduction in protein levels after FLAG or myc insertion in the transmembrane domain, full-length constructs were made for the FLAG epitope in loop 2 and 3, and the SA-1 insertion in loop 1. These constructs were transformed to LMC500 and MC1061 ΔOmpA and were subjected to the same induction experiment as for the OmpA TM domain variants. Without induction, all full-length constructs grew normally in both genetic backgrounds. After induction, the growth rate remained unaffected for all full-length OmpA loop insertion constructs in the MC1061 ΔOmpA background whereas expression in LMC500 resulted in growth curves similar to those obtained for the OmpA-177 constructs (only tested for the loop 3 FLAG insertion).

**Figure 4:**
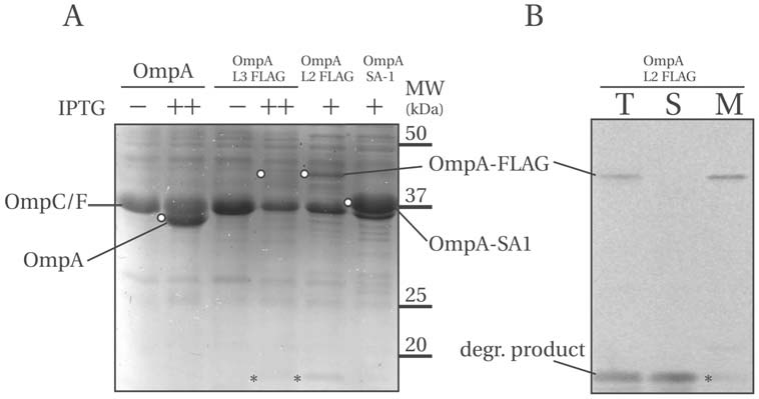
The periplasmic domain does not stabilize the TM domain and FLAG constructs are degraded. Left panel: Coomassie stained 15% SDS-PAGE. Membrane fractions corresponding to 1 ml OD_600_ of 0.625 cells were loaded. IPTG was either absent (−), or added during exponential growth at an end concentration of 0.3 mM (+), or 1 mM (++). Envelopes were isolated from MC1061ΔOmpA. Open circles indicate the position of the expressed constructs. The position of the OmpC/F band is indicated. The asterisks indicate a degradation band that reacted with anti-OmpA. Right panel: full-length OmpA with a loop 2 FLAG insertion induced with 0.3 mM IPTG, fractionated in total, soluble and membrane fraction. The immunoblot was probed with anti-OmpA (1∶10000). The large degradation band at around 17 kDa is clearly visible, and was almost completely soluble. The strain was MC1061ΔOmpA.

The full-length constructs were detected both on Coomassie stained gel (Figure 4A) as well as on immunoblot using a polyclonal antibody that recognizes the periplasmic domain of OmpA (Figure 4B, Figure S4). As with the FLAG OmpA-177 proteins, full-length OmpA FLAG constructs had a higher apparent molecular weight than calculated. Comparing soluble to membrane fractions using immunoblots yielded similar results as for the OmpA-177 variants, with the majority of each construct fractionating to the membrane fraction, except loop 3, which was divided over the soluble and membrane fraction (Figure S4).

**Figure 5:**
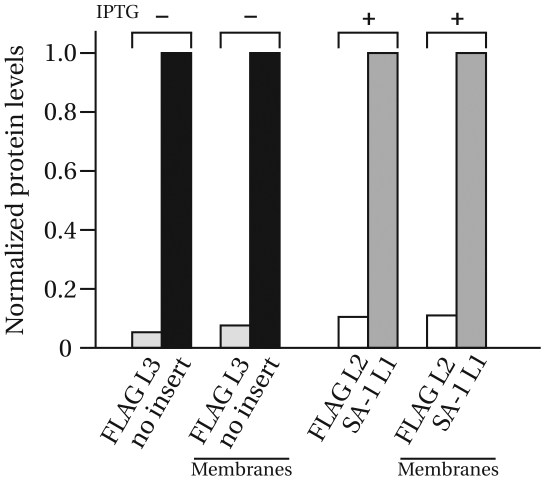
Comparison of protein levels of OmpA with and without inserted peptides. FLAG insertions, either in Loop 2 or 3, are present at approx. 5–10% relative to either the full-length OmpA without insertion or the full-length OmpA with a SA-1 insertion in Loop 1. The levels were quantified from immunoblots by densitometry using ImageJ and are normalized relative to the stronger band. Both total cell lysates and membrane fraction were quantified. For 3xFLAG in Loop 3, compared with full-length OmpA without insertion, no IPTG was added. For 3xFLAG in Loop 2, compared with SA-1 in Loop 1, constructs were induced with 0.3 mM IPTG.

Again, the FLAG constructs appear greatly reduced compared to both the OmpA without insertion and the OmpA with an SA-1 insertion, all expressed from plasmid. The intensities of the anti-OmpA bands from Figure S4 were quantified using ImageJ and plotted as bar graphs in Figure 5. Together with Figure 4A, it can be concluded that full-length OmpA constructs with FLAG insertions either in loop 2 or loop 3, are present at approximately 5–10% compared to full-length OmpA without insertion or the full-length OmpA carrying a SA-1 insertion, respectively.

Apart from intact construct, the immunoblots probed with anti-OmpA antibody revealed a strong band around 17 kDa (the expected size of the periplasmic domain) that fractionates to the soluble fraction (Figure 4B). This band was absent from samples containing induced full-length OmpA without insertion and weakly detected in induced full-length OmpA with the SA-1 insertion (data not shown). From this we conclude that the degradation band is specific and that it is related to the reduced protein levels of the FLAG constructs. In addition, the FLAG constructs were detected using anti-FLAG antibody (Figure S4 and not shown). The 17 kDa degradation band did not react with anti-FLAG (the smallest degradation band detected with anti-FLAG ran around 27 kDa). Apparently, the periplasmic domain of OmpA was cleaved from the TM domain and the latter was to a large extent degraded.

To test whether the 17 kDa degradation band was present in periplasmic aggregates, we fractionated the cells into soluble periplasmic contents and insoluble pellet by osmotic shock (See Figure S6 and Materials and Methods S1). We find that the degradation band was not aggregated but present in the soluble periplasmic fraction, consistent with the idea that the FLAG/myc OmpA variants are to a large extent degraded by periplasmic proteases.

Taken together, it can be concluded that both the full-length and the OmpA-177 constructs with a FLAG insertion are present in greatly reduced amounts, compared to without insertion or with a SA-1 insertion. Therefore, addition of the periplasmic domain does not improve protein levels of the FLAG insertion. Furthermore, the reduction in protein levels is, at least partly, caused by degradation, as observed on immunoblots.

### OM incorporation of truncate and full-length OmpA constructs

To study whether the OmpA variants, present in the membrane fraction, have obtained their native form, we examined their heat-modifiability. The native form of the OmpA TM domain is a compact β-barrel that has a particularly tight fold with a half life of 30 min when heated to 72°C in 2% SDS [23]. When heated in SDS at lower temperatures (e.g. 50°C) the β-barrel becomes soluble without unfolding and migrates faster through the gel (30 kDa) relative to its denatured form, which runs at the expected molecular weight (35 kDa). This effect is called heat modifiability [14] and it is a general property of β-barrels. Various techniques have been used to confirm that the 30 kDa form corresponds to the native fold of OmpA (for references consult [24]. It is generally assumed that *in vivo*, the native form of OmpA is generated only after proper insertion into the outer membrane (Ried, 1994).

**Figure 6:**
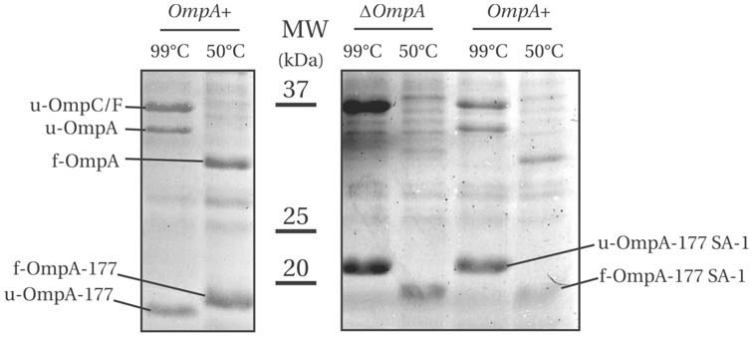
The OmpA TM domain constructs OmpA-177 and OmpA-177 SA-1 are fully heat modifiable. A Coomassie stained gel containing membrane fractions (corresponding to 1 ml OD_600_ of 0.625 cells) from cells expressing either OmpA-177 or OmpA-177 SA-1 constructs induced with 1 mM (OmpA-177) or 0.3 mM (OmpA-177 SA-1) IPTG, either in LMC500 (OmpA+) or MC1061 ΔOmpA. The samples were either heated in sample buffer for 5 min at 99°C or for 15 min at 50°C. Folded proteins are prefixed with f- and unfolded proteins are prefixed with u-.

Cell membranes containing IPTG-induced OmpA-177 or OmpA-177 SA-1 proteins, expressed in LMC500 or MC1061 ΔOmpA were either heated in sample buffer for 5 min at 99°C or for 15 min at 50°C, then applied on gel, separated by SDS-PAGE and stained with Coomassie (Figure 6). Note that both OmpC and OmpF do not become soluble at 50°C and are therefore only visible in samples heated at 99°C [10]. As expected, the endogenous OmpA of LMC500 is fully heat-modifiable. Furthermore, the OmpA-177 protein, but not the OmpA-177 containing the SA-1 insertion, shows the aberrant heat-modifiability already observed in the literature for OmpA-171 [10], where the folded protein migrates slower than the unfolded protein (see also discussion). Finally, the OmpA-177 SA-1 protein is also fully heat-modifiable. We conclude that the majority, if not all, of both constructs have reached their native form.

**Figure 7:**
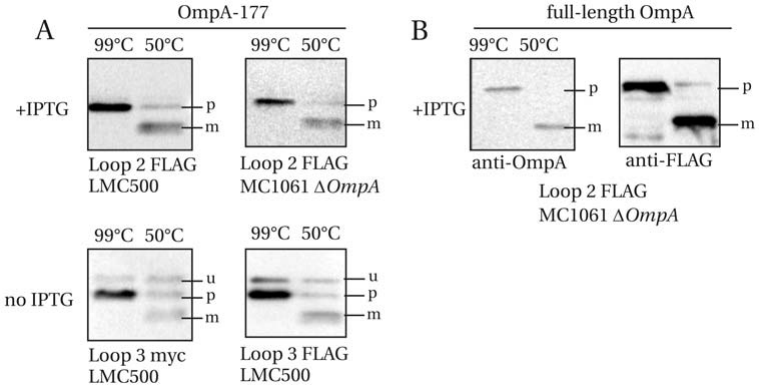
OmpA-177 domain variants with FLAG or myc epitopes and a full-length OmpA variant with a FLAG epitope are predominantly heat-modifiable. Cell envelopes of various OmpA-177 TM domain variants (A) and the full-length Loop 2 FLAG construct (B) were either heated in sample buffer for 5 min at 99°C or for 15 min at 50°C, before being separated on a 15% SDS-PAGE and immunoblotted. Indicated are: (p) processed, unfolded, (m) matured, properly folded and (u) unprocessed, unfolded. In (A) 0.1 µg/ml anti-FLAG was used for the induced Loop 2 construct, and 1 µg/ml anti-myc and 0.5 µg/ml anti-FLAG were used for the uninduced Loop 3 constructs. In (B), 1∶10000 anti-OmpA, and 0.1 µg/ml anti-FLAG was used.

For the FLAG and myc constructs, because of their low levels in the cell, immunoblotting was used to visualize their heat-modifiability (Figure 7). The OmpA TM domain constructs with FLAG or myc are all predominantly heat-modifiable (Figure 7A, indicated “mature”). As expected, the loop 3 precursor bands (indicated “unprocessed”), present in the membrane fraction, are not at all heat-modifiable. Also a full-length OmpA construct carrying a loop 2 FLAG insertion was found to be fully heat-modifiable (Figure 7B). We conclude that all FLAG and myc constructs are at least partially heat-modifiable, and thus can be properly incorporated in the outer membrane.

### Surface display of loop insertions: fluorescent labeling of cells

To determine the accessibility on the cell surface of the inserted antigenic peptides, both fixed and living cells were labeled. Cells carrying the FLAG and myc in either loop 2 or 3 of OmpA-177 were induced with 0.3 mM IPTG, fixed and stained with monoclonal anti-FLAG or monoclonal anti-myc. As a negative control, the primary antibody was left out, and no fluorescence was observed for any of these samples. All four constructs were detected on the bacterial cell surface (Figure S5). Loop 2 insertions show more staining at the poles, whereas loop 3 insertions are more homogeneous.

Living cells were labeled using a biotinylated variant of the anti-FLAG antibody. Labeling of living cells was performed because it was found that fluorescent streptavidin (needed for the SA-1 peptide) penetrated fixed cells to show a nucleoid-like staining (data not shown). The cells could be fixed after labeling to preserve the staining.

**Figure 8:**
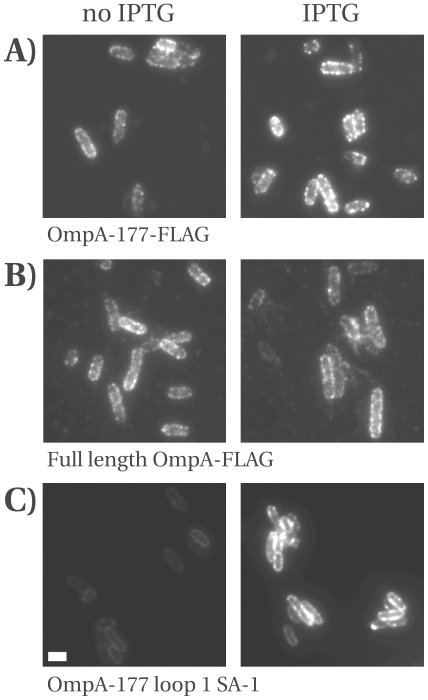
Detection of the inserted peptides on the cell surface. Fluorescently labeled cells expressing (A) FLAG in loop 3 of OmpA-177, (B) FLAG in loop 3 of OmpA (full-length), or (C) SA-1 in loop 1 of OmpA-177. Cells were grown in TY medium either without IPTG or with 0.3 mM IPTG added and induced for 2 hours before labeling. Exposure times: (A, B) 1 s, (C) 150 ms. Strain was LMC500. Scale bar dimensions are 1 by 2 µm.

Results of labeled, uninduced cells carrying either OmpA-177 FLAG (loop 3) or the full-length OmpA-FLAG (loop 3) showed that both have comparable levels of antibody-accessible FLAG epitope on their surface (Figure 8A, B). This provides further evidence that adding the periplasmic domain does not result in increased stability of the protein. The limited increase in fluorescence after induction correlates with a modest increase of intact protein, and a larger increase of the 17 kDa degradation band as detected on immunoblot (data not shown).

Finally, living LMC500 cells expressing OmpA-177-SA1 were directly labeled with fluorescent streptavidin, fixed and imaged (Figure 8C). To be able to compare with and without induction directly, a short exposure time (150 ms) was chosen. Without induction staining was homogeneous along the perimeter of the cells. After induction (and after recovery from the lag phase that occurs in LMC500, see earlier), fluorescence increased markedly, and during labeling, strong streptavidin-mediated cross-linking between cells occurred, leading to clumps. Overall, we conclude that all the constructs are detected on the cell surface, but for FLAG, and likely also myc, the increase after induction is reduced due to degradation, whereas for SA-1, both efficient labeling and a strong effect of induction on the surface display is observed.

## Discussion

In this work we have characterized peptide insertions in the OmpA protein, both in TM domain constructs (OmpA-177) and full-length constructs. As insertions we used the popular FLAG and myc epitopes, and a streptavidin binding peptide SA-1. It has been demonstrated that similar-sized peptides can be inserted in loop 2 of OmpA without reduction in protein levels or membrane incorporation [3], [18]. Unexpectedly, introducing a FLAG or myc peptide at this location in the OmpA protein reduced the protein levels with approximately 90% (Figure 3, Figure 5). However, the majority of the intact protein was inserted properly in the OM, as judged by its heat-modifiability (Figure 7A). In contrast, insertion of the SA-1 peptide in loop 1 did not reduce protein levels, similar to the reported insertions in loop 2 (Table 2). These results have been summarized in Table 4.

**Table 4:** Comparison of similar-sized peptide tags inserted in OmpA.

Peptide	Insertion size (AA)	Protein levels (%)^a^	Proper OM incorporation^b^	Surface display peptide^c^
None	0	100	yes	NA
3xFLAG	22	5–10	yes	yes
2xmyc	20	5–10	yes	yes
SA-1	15	90–100	yes	yes

a Relative protein levels compared to OmpA without tag inserted (first row in this table, set at 100%).

b Proper OM incorporation deduced from proper β-barrel formation using the heat-modifiability assay.

c Surface display of the inserted tag deduced from fluorescent labeling of intact cells.

It could be argued that the observed differences are due to over-expression of the proteins. However, our expression vector (a weakened pTrc99A [16]) produces less than 5⋅10^3^ proteins in the absence of IPTG [25], and already at these low expression levels the marked difference in protein level between OmpA with a FLAG insertion and OmpA without insertion is observed (Figure 5). This suggests that introducing the FLAG epitope leads to an intrinsically reduced protein level, independent of the induction level.

What could be the reason of the observed reduction in protein levels of the FLAG and myc constructs? Both epitopes are highly charged (Table 2). In addition, both the FLAG and myc epitopes are effectively negatively charged, and therefore might interact unfavorably with the negatively charged LPS in the OM, or with the YaeT/BamA protein complex responsible for OM insertion [26]. We speculate that a reduced rate of OM incorporation might cause a buildup of unincorporated (misfolded) OmpA proteins, whose subsequent degradation would explain the reduced cellular levels of the FLAG and myc constructs reported here.

Although the number of tested insertions thus far is small, one might speculate that the abundant charge of the inserted epitopes is the cause of the degradation and consequently, of the reduced display levels. Negative effects on OM incorporation after insertion of highly charged peptides have also been reported for outer membrane protein PhoE [27]. It was found that an 20-mer insertion containing 10 positively charged residues, as well as a 28-mer insertion containing 6 negative and 2 positively charged residues were not correctly incorporated in the OM [27], whereas a PhoE variant with a VP1 epitope inserted at the same location was incorporated in the OM at normal protein levels [28], [29]. It might thus be worth testing more systematically the effect of charged peptide insertions on OMP incorporation in systems that are used for display and/or screening purposes.

One may subsequently ask to what extent differences in display levels affect the application of the OmpA scaffold in, for instance, random library screening using FACS. Whether variations in display efficiency affect a particular screening method is determined by the detection sensitivity and specificity of that method. Interestingly, the FLAG anti-FLAG M2 epitope/antibody combination has been subjected to epitope mapping with MACS and FACS using the related OmpX display system, and the consensus recognition sequence DYKDxD [30], [31] was successfully recovered [4]. This indicates that selection for a sequence containing four charged residues in a surface-displayed library of random 15-mers is possible. Unfortunately, whether the isolated clones had reduced display levels was not determined. It will be interesting to assess if peptides with more (mostly negative) charge such as 3xFLAG or 2xmyc can also be selected for with FACS, and to what extent the various selection methods are affected by display biases.

Unexpectedly, in exponentially growing cells without IPTG, about 25% of the loop 3 insertions are not processed. Since OmpA is mostly post-translationally translocated to the periplasm via the Sec system [32], in these cases perhaps some tertiary structure forms in the cytoplasm that delays, or interferes with, its translocation [33]. However, induction of constructs with FLAG in loop 2 or loop 3 results in similar amounts of processed OmpA (Figure S2), and in similar amounts of FLAG epitope detected on the bacterial surface (Figure S5). This suggests that after processing and release into the periplasm, both proteins behave in a similar way, and we conclude that the adverse effects of the FLAG and myc epitopes on protein levels do not appear to be loop specific.

Surprisingly, induction of either (FLAG or myc) loop 2 constructs or the OmpA-177-SA1 in LMC500 (MC4100 *lysA*) affects growth rate profoundly in a transient manner. It is difficult to understand that loop 2 FLAG/myc and loop 1 SA-1 constructs cause a similar effect on growth rate in LMC500, since their amounts differ 10-fold. Perhaps the cell regulates the amount of proteins in the OM that are tolerated (little in the case of FLAG/myc, a lot in the case of SA-1). When IPTG induction disturbs this balance, the observed lag period of 30 min might reflect a period in which the cell adapts and restore this balance, after which the cells continue growth.

Accumulation of misfolded OMPs in the cell envelope causes the activation of the σ^E^ controlled extracytoplasmic stress response [34] that down-regulates OMP expression ([35]). Indirect evidence for σ^E^ activation upon IPTG induction of our OmpA loop insertion variants comes from the membrane fractions shown in Figures 3 and 4, where OMP expression (OmpC/F and OmpA) is consistently down-regulated after IPTG induction, but only for loop insertion variants.

In the different genetic background of strain MC1061 and its derivative MC1061 ΔOmpA, expression levels were similar to LMC500, but the growth rate was unaffected upon IPTG induction (data not shown). It has been shown that the σ^E^ transcription factor is also controlled by intracellular ppGpp levels [36]. MC1061 has the *spoT1* mutation, that abolishes the ppGppase activity of SpoT and results in increased levels of (p)ppGpp (Cashel, 1996). This could offer an explanation for the observed robustness of MC1061 towards overexpression of the OmpA loop insertion constructs. If MC1061 is better able to cope with folding stress in the periplasm (e.g. by having its stress response genes already expressed, or in higher levels), the balance can be restored immediately, without disturbing the growth rate. Whatever the molecular mechanism may be, our results indicate that MC1061 is a strain of choice when over-expressing engineered outer membrane proteins such as OmpA.

Finally, our results provide insight on the aberrant heat-modifiability observed for 8-stranded β-barrels such as OmpA-171, OmpA-177 and NspA ([10], [37] and this study). Heat-modifiability has been termed “aberrant” when the unfolded form migrates faster through the gel compared to the folded form. Surprisingly, for the OmpA-177 insertion variants (SA-1, FLAG or myc) heat-modifiability is “normal” again (Figure 6). Comparing the mobility of OmpA-177 to OmpA-177-SA-1, we find that the folded barrels run at almost similar height, as if the extra residues of SA-1 were absent, whereas after boiling, OmpA-177-SA-1 is retarded with respect to OmpA-177 with an amount corresponding to their difference in molecular mass. In other words, the additional residues inserted in the exterior loop have a stronger impact on the unfolded form than on the folded form. These results suggest that as more and more residues are added to the OmpA-171 TM domain, the relative positions between the folded and unfolded domains first decrease until they are equal, before increasing again to appear as “normal” heat-modifiability. This predicts that for some rare OMPs, it would seem as if they would not have any heat-modifiability at all.

### Conclusions

Most bacterial display studies focus on either identifying novel scaffolds or novel applications. In this work, a thorough analysis was performed of constructs that contain similar-sized peptides inserted into the OmpA display scaffold. The effect of their expression on *E. coli* growth, as well as their relative amount, sub-cellular localization, folding state and surface accessibility has been determined. In doing so, we believe that several useful observations have been made that are relevant for the fields of surface display, (outer) membrane protein over-expression and purification as well as for applications that make use of bacteria as antigen-displaying whole-cell adsorbents.

First, we conclude that OmpA displays not all small peptides equally efficiently, since the highly charged and hydrophilic 3xFLAG and 2xmyc epitopes reduce protein levels 10-fold due to degradation by periplasmic proteases. Second, the growth curves obtained after inducing high-level expression of the OmpA epitope variants in two standard laboratory strains MC4100 (*LysA*) and MC1061 suggest that strain MC1061 is a strain of choice when expressing OMPs in large amounts in *E. coli*. Finally, our observation that the heat modifiability of the OmpA TM domain can be changed from ‘aberrant’ to normal by insertion of small epitopes predicts that some β-barrels might not exhibit significant heat-modifiability at all.

## Supporting Information

Figure S1 Predicted protein structures by SWISS-MODEL of the OmpA transmembrane domain before and after epitope insertion. PDB entries 1g90.pdb, 1bxw.pdb and 1qjp.pdb were used to build the model. (A) OmpA-177 model. (B) OmpA-177 model with 2xmyc inserted in loop 2 after G70. (C) OmpA-177 model with 3xFLAG inserted in loop 3 after N109.
(1.28 MB TIF)

Figure S2 Detection of OmpA-177 TM domain variants with inserted 3xFLAG or 2xmyc peptides on immunoblot. Expression of the variants was induced in LMC500 with 0.3 mM IPTG. From left to right: OmpA-177 loop 2 myc, OmpA-177 loop 2 FLAG, OmpA-177 loop 3 myc and OmpA-177 loop 3 FLAG. For loop 3 variants, unprocessed protein is also present. Left panel: anti-FLAG (3 µg/ml), right panel: anti-myc (3 µg/ml). For this blot, a 12% SDS-PAGE gel percentage was used.
(0.82 MB TIF)

Figure S3 The OmpA TM domain constructs are predominantly present in the membrane fraction. Total cell lysate (T) was fractionated into soluble (S) and membrane (M) fractions. Shown are immunoblots of constructs OmpA-177 loop 2 FLAG (induced), OmpA-177 loop 3 myc (uninduced), and OmpA-177 loop 3 FLAG (uninduced). Strain is LMC500, except for loop 2 FLAG, where results from strain LMC500 and MC1061ΔOmpA are shown. Only the relevant portions of the blot are shown. Black line indicates 25 kDa marker band. Antibody concentrations used were 1 µg/ml (anti-myc), and 0.1 µg/ml or 0.5 µg/ml (anti-FLAG) for induced or uninduced FLAG, respectively.
(0.42 MB TIF)

Figure S4 The full-length OmpA constructs (except loop 3 FLAG) fractionate predominantly to the membrane fraction. Total cell lysate (T) was fractionated into soluble (S) and membrane (M) fractions. Shown are immunoblots of full-length OmpA constructs carrying a FLAG insertion in loop 2 (d) or loop 3 (c), and an SA-1 insertion in loop 1 (e). Strain was MC1061ΔOmpA. As controls, fractions of LMC500 (endogenous OmpA, OmpA+) (a), and OmpA expressed from plasmid in MC1061ΔOmpA (b) are shown. Only the relevant portions of the blot are shown. Black line indicates 37 kDa marker band. For the wild type OmpA and induced constructs, a 1∶10000 dilution was used for the polyclonal antibody against OmpA. For the uninduced construct, a 1∶1000 dilution was used. Anti-FLAG was used for the induced and uninduced FLAG constructs at 0.1 µg/ml and 1 µg/ml, respectively. Band intensities in the anti-OmpA blots (b) and (c), and (d) and (e) can be compared directly. Their relative intensities, quantified using densitometry with ImageJ, are shown in Fig. 5.
(1.10 MB TIF)

Figure S5 Myc and FLAG epitopes are detected on the surface of cells expressing OmpA-177 TM domain variants. Cells induced with 0.3 mM IPTG for expression of OmpA-177 containing either FLAG in loop 2, myc in loop 2, FLAG in loop 3 or myc in loop 3, were fixed and immuno-labeled with antibodies against FLAG or myc. The scale bar corresponds to 2 µm. Image exposure time was 470 ms.
(2.56 MB TIF)

Figure S6 The 17 kDa OmpA degradation band fractionates to the soluble periplasmic fraction. Soluble periplasmic fractions (s) and insoluble cell pellet (i) were prepared as described in Supplementary Materials and Methods. Shown are immunoblots of the strain MC1061ΔOmpA containing constructs expressing full-length OmpA without tag insertion (pGI9) and full length OmpA with a 3xFLAG inserted in Loop 2 (pGV32). The latter protein is present at the expected height in the insoluble fraction, as expected for a properly assembled OM protein. The stronger 17 kDa degradation band is present in the soluble periplasmic fraction instead. Only the relevant portions of the blot are shown. The soluble periplasmic protein MBP (40 kDa), inducible by growth on maltose (0.2%), was used as a fractionation marker. The polyclonal anti-OmpA antibody was used in a 1∶10000 dilution (upper blot) and the monoclonal anti-MBP antibody (Abcam, #ab65) was used at a concentration of 1.8 µg/ml (lower blot).
(1.06 MB TIF)

Materials and Methods S1 Contains detailed plasmid cloning steps and the fractionation protocol for Figure S6.
(0.03 MB DOC)
