# Supplementary figures and images for: Differential Bacterial Surface Display of Peptides by the Transmembrane Domain of OmpA
Source: PLoS One. 2009 Aug 25;4(8):e6739. doi: 10.1371/journal.pone.0006739 (PMC2726941; doi:10.1371/journal.pone.0006739)

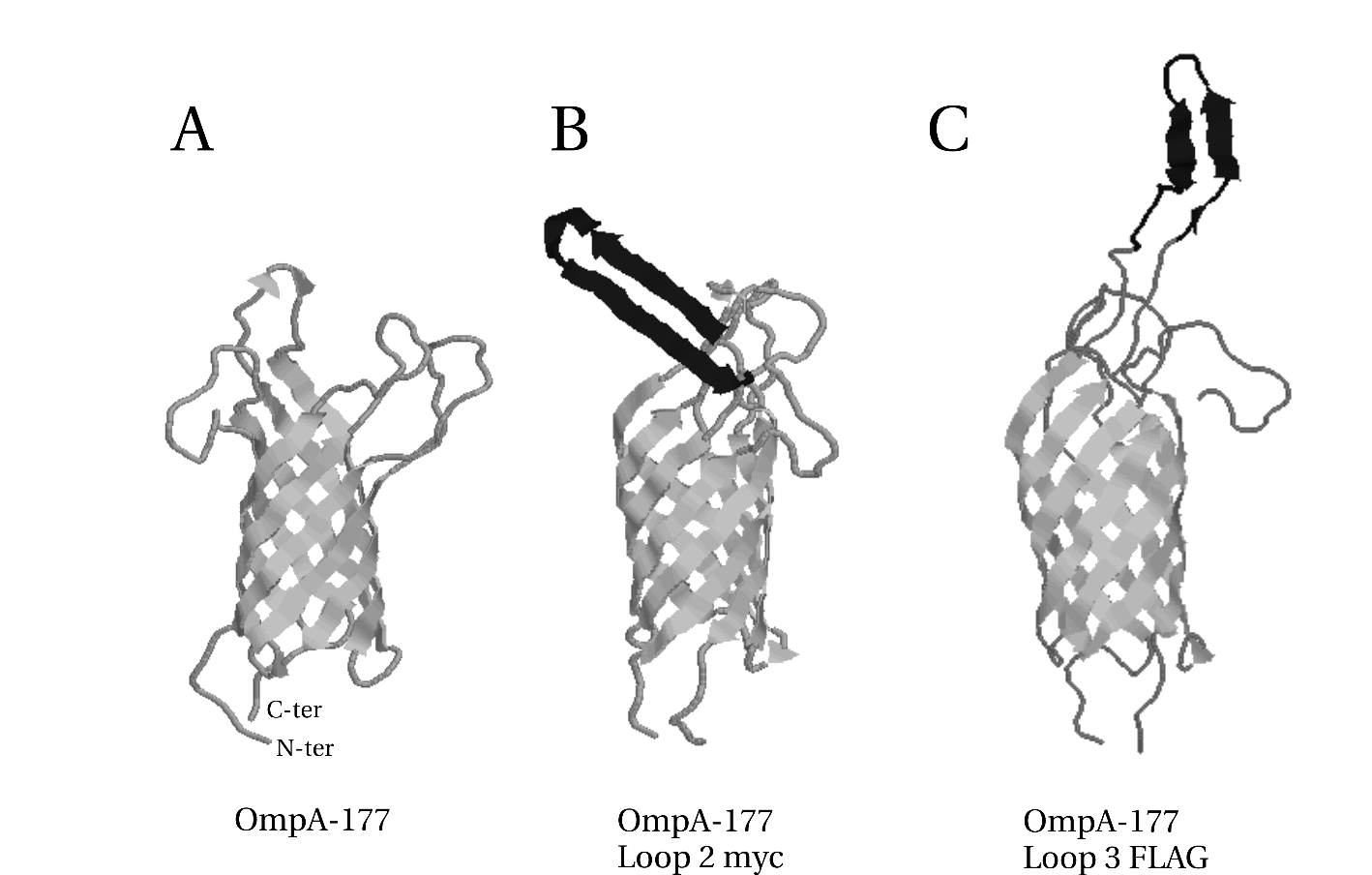

Supplement: Figure S1 — Predicted protein structures by SWISS-MODEL of the OmpA transmembrane domain before and after epitope insertion. PDB entries 1g90.pdb, 1bxw.pdb and 1qjp.pdb were used to build the model. (A) OmpA-177 model. (B) OmpA-177 model with 2xmyc inserted in loop 2 after G70. (C) OmpA-177 model with 3xFLAG inserted in loop 3 after N109. (1.28 MB TIF) [file pone.0006739.s001.tif]

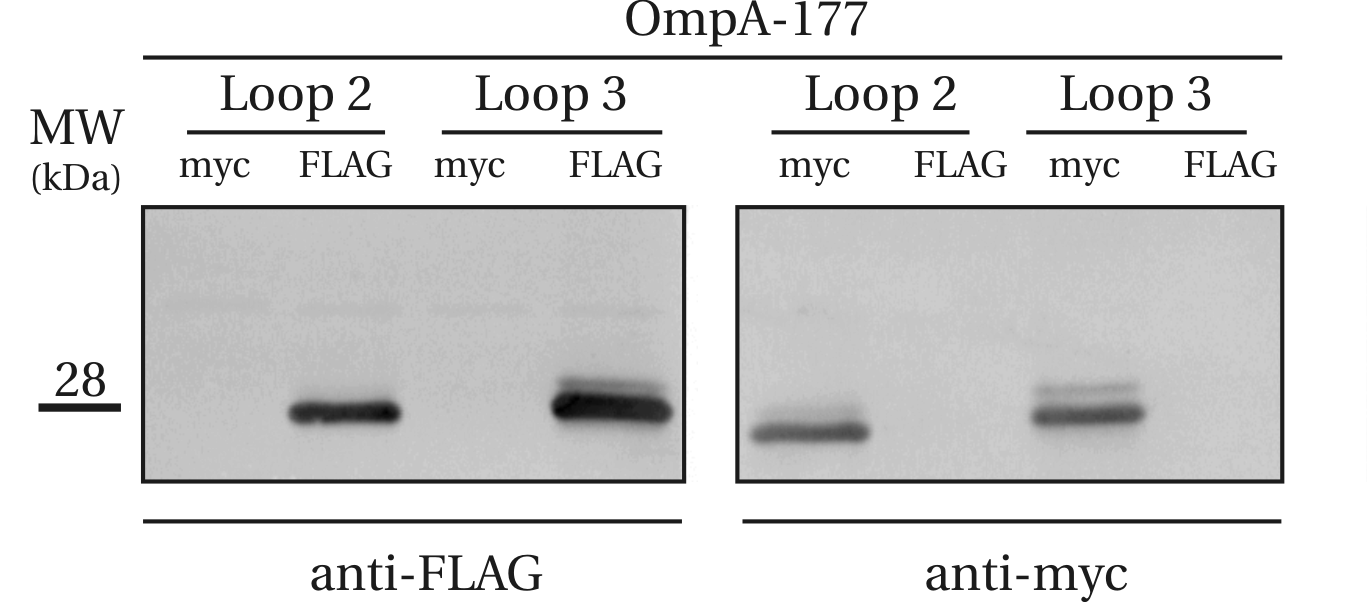

Supplement: Figure S2 — Detection of OmpA-177 TM domain variants with inserted 3xFLAG or 2xmyc peptides on immunoblot. Expression of the variants was induced in LMC500 with 0.3 mM IPTG. From left to right: OmpA-177 loop 2 myc, OmpA-177 loop 2 FLAG, OmpA-177 loop 3 myc and OmpA-177 loop 3 FLAG. For loop 3 variants, unprocessed protein is also present. Left panel: anti-FLAG (3 µg/ml), right panel: anti-myc (3 µg/ml). For this blot, a 12% SDS-PAGE gel percentage was used. (0.82 MB TIF) [file pone.0006739.s002.tif]

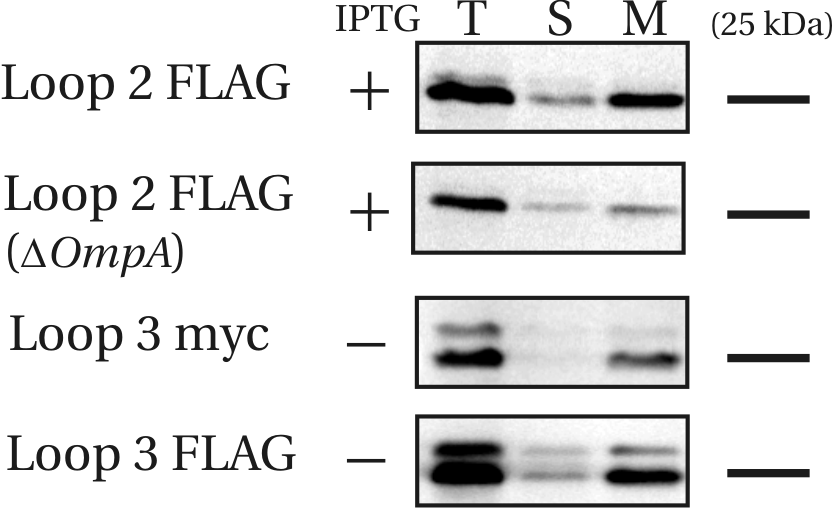

Supplement: Figure S3 — The OmpA TM domain constructs are predominantly present in the membrane fraction. Total cell lysate (T) was fractionated into soluble (S) and membrane (M) fractions. Shown are immunoblots of constructs OmpA-177 loop 2 FLAG (induced), OmpA-177 loop 3 myc (uninduced), and OmpA-177 loop 3 FLAG (uninduced). Strain is LMC500, except for loop 2 FLAG, where results from strain LMC500 and MC1061ΔOmpA are shown. Only the relevant portions of the blot are shown. Black line indicates 25 kDa marker band. Antibody concentrations used were 1 µg/ml (anti-myc), and 0.1 µg/ml or 0.5 µg/ml (anti-FLAG) for induced or uninduced FLAG, respectively. (0.42 MB TIF) [file pone.0006739.s003.tif]

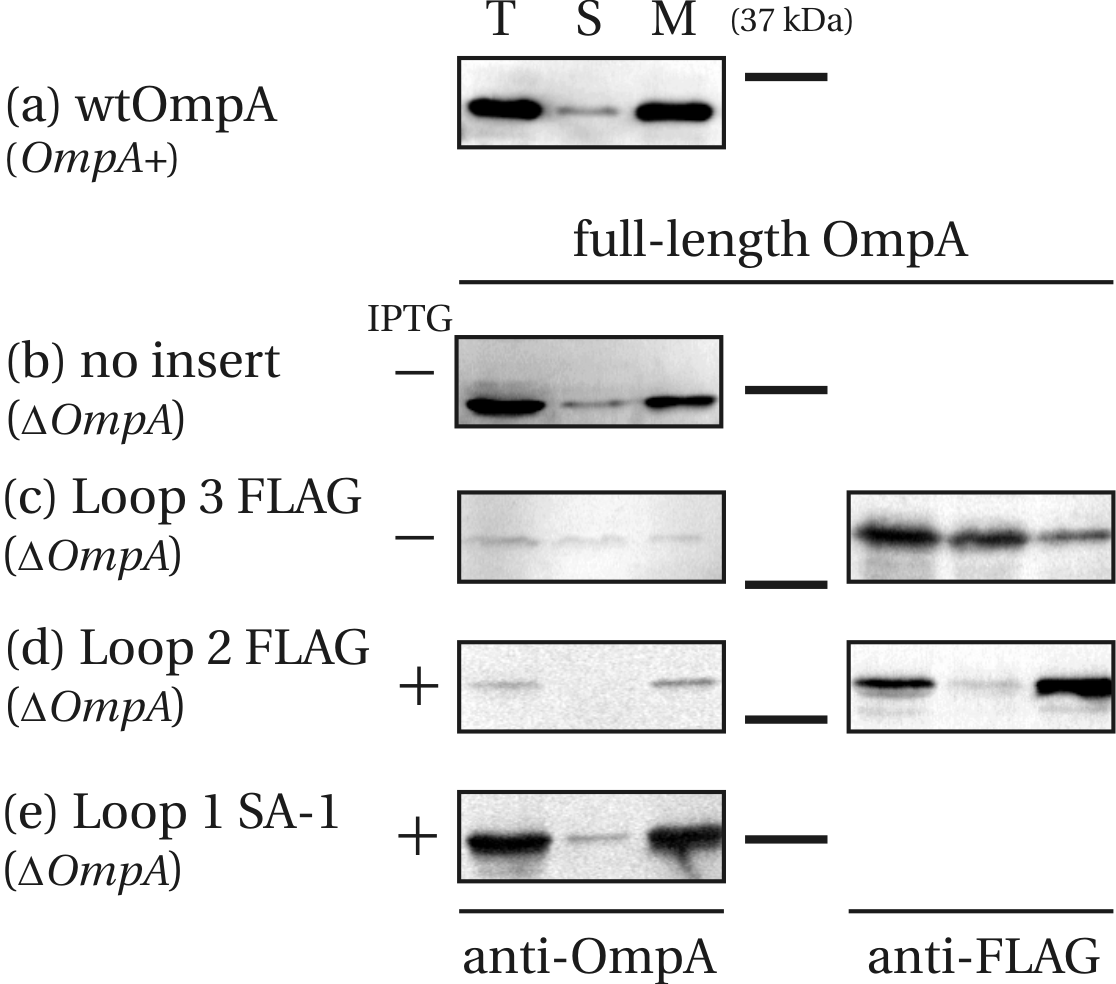

Supplement: Figure S4 — The full-length OmpA constructs (except loop 3 FLAG) fractionate predominantly to the membrane fraction. Total cell lysate (T) was fractionated into soluble (S) and membrane (M) fractions. Shown are immunoblots of full-length OmpA constructs carrying a FLAG insertion in loop 2 (d) or loop 3 (c), and an SA-1 insertion in loop 1 (e). Strain was MC1061ΔOmpA. As controls, fractions of LMC500 (endogenous OmpA, OmpA+) (a), and OmpA expressed from plasmid in MC1061ΔOmpA (b) are shown. Only the relevant portions of the blot are shown. Black line indicates 37 kDa marker band. For the wild type OmpA and induced constructs, a 1∶10000 dilution was used for the polyclonal antibody against OmpA. For the uninduced construct, a 1∶1000 dilution was used. Anti-FLAG was used for the induced and uninduced FLAG constructs at 0.1 µg/ml and 1 µg/ml, respectively. Band intensities in the anti-OmpA blots (b) and (c), and (d) and (e) can be compared directly. Their relative intensities, quantified using densitometry with ImageJ, are shown in Fig. 5. (1.10 MB TIF) [file pone.0006739.s004.tif]

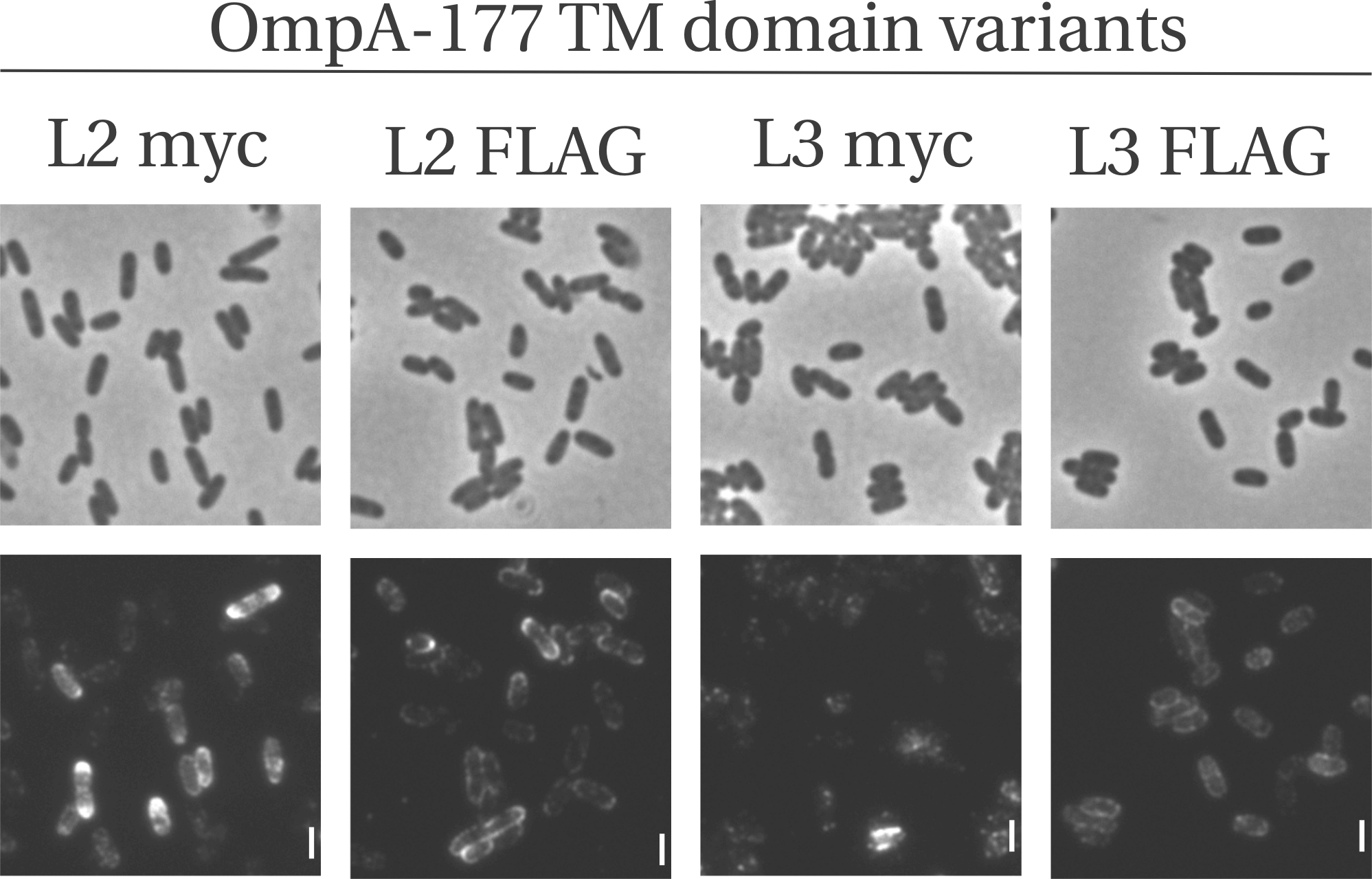

Supplement: Figure S5 — Myc and FLAG epitopes are detected on the surface of cells expressing OmpA-177 TM domain variants. Cells induced with 0.3 mM IPTG for expression of OmpA-177 containing either FLAG in loop 2, myc in loop 2, FLAG in loop 3 or myc in loop 3, were fixed and immuno-labeled with antibodies against FLAG or myc. The scale bar corresponds to 2 µm. Image exposure time was 470 ms. (2.56 MB TIF) [file pone.0006739.s005.tif]

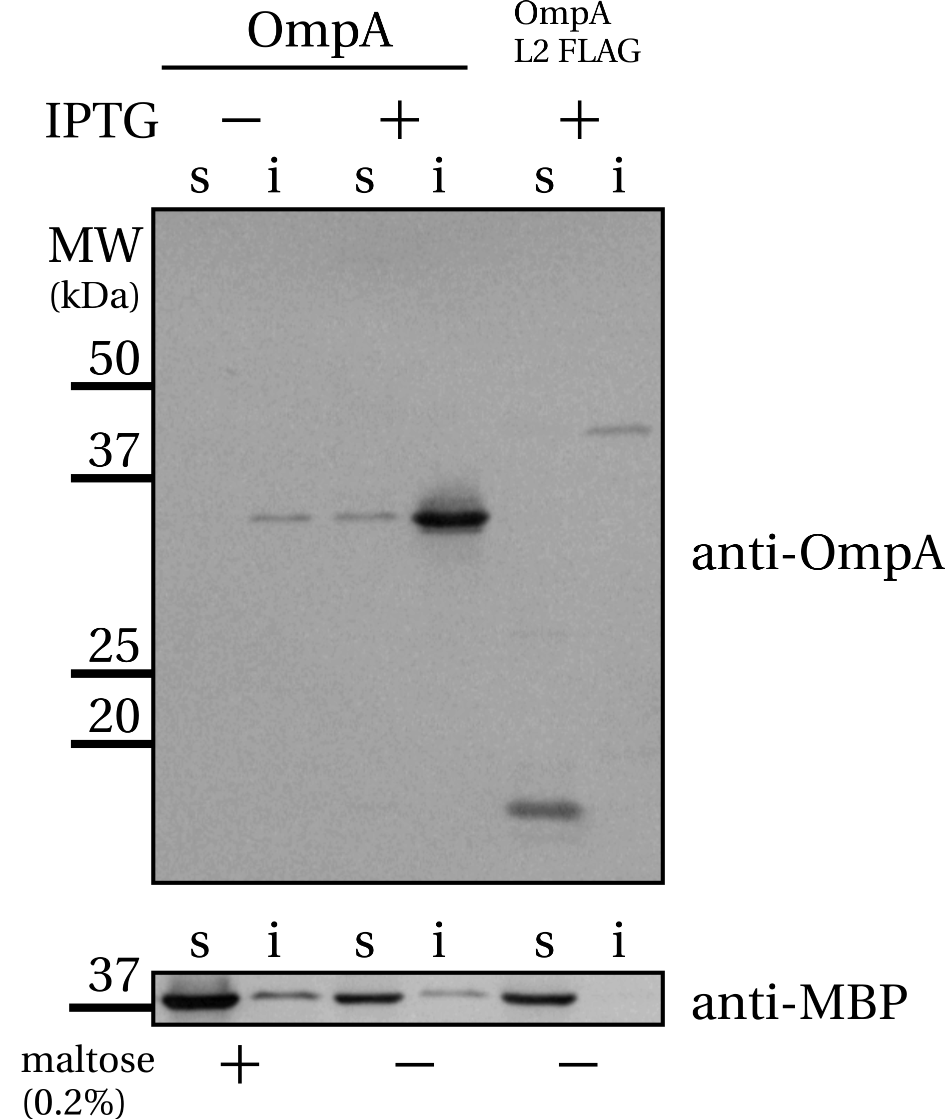

Supplement: Figure S6 — The 17 kDa OmpA degradation band fractionates to the soluble periplasmic fraction. Soluble periplasmic fractions (s) and insoluble cell pellet (i) were prepared as described in Supplementary Materials and Methods. Shown are immunoblots of the strain MC1061ΔOmpA containing constructs expressing full-length OmpA without tag insertion (pGI9) and full length OmpA with a 3xFLAG inserted in Loop 2 (pGV32). The latter protein is present at the expected height in the insoluble fraction, as expected for a properly assembled OM protein. The stronger 17 kDa degradation band is present in the soluble periplasmic fraction instead. Only the relevant portions of the blot are shown. The soluble periplasmic protein MBP (40 kDa), inducible by growth on maltose (0.2%), was used as a fractionation marker. The polyclonal anti-OmpA antibody was used in a 1∶10000 dilution (upper blot) and the monoclonal anti-MBP antibody (Abcam, #ab65) was used at a concentration of 1.8 µg/ml (lower blot). (1.06 MB TIF) [file pone.0006739.s006.tif]
