# Supplementary material for: Differential Bacterial Surface Display of Peptides by the Transmembrane Domain of OmpA
Source: PLoS One. 2009 Aug 25;4(8):e6739. doi: 10.1371/journal.pone.0006739 (PMC2726941; doi:10.1371/journal.pone.0006739)
Supplement: Materials and Methods S1 — Contains detailed plasmid cloning steps and the fractionation protocol for Figure S6. (0.03 MB DOC) [file pone.0006739.s007.doc]

# Supplementary Materials and Methods

### DNA cloning steps

The signal sequence and the first 177 residues of the mature OmpA protein, coding for the transmembrane domain, were cloned in the expression vector pTrc99A [1], modified to decrease the basal expression level (i.e. without inducer) to typically a few thousands proteins per cell (pTHV037, [2]). OmpA-177 was amplified by PCR from the chromosome of LMC500 using primers proOmpANcoIfw (5-CGGCAGCCATGGCAAAAAAGACAGCTATCGCG-3) and OmpAHindIIIrv (5-CCTGGCTAAGCTTATGGAGCTGCTTCGCCC-3), and ligated in the *Nco*I and *Hind*III sites of pTHV037 to create pMD5. The *Nco*I site introduces after the start Met codon an additional alanine codon. The 3xFLAG and 2xmyc epitope loop insertions in loop 2 and 3 (constructs pGV1-4, see Table 1) were created using overlap PCR. For instance, to create the 3xFLAG insertion in loop 2 of the OmpA TM domain (pGV2), two separate PCRs, containing a region of overlap, were performed on pMD005. The first PCR with primers proOmpANcoIfw and 3xflagOmpAL2RV(5-ATCGATGTCATGATCTTTATAATCACCGTCATGGTCTTTGTAGTCACCGTTTTCAACGCTGCCTTTGTAC-3), and the second PCR with primers 3xflagOmpAL2FW (5-GATTATAAAGATCATGACATCGATTACAAGGATGACGATGACAAGGCATACAAAGCTCAGGGCGTTCAAC-3) and OmpAHindIIIrv. The two PCR fragments were then mixed, denatured and annealed to form a duplex at the overlap region, and filled in by DNA polymerase (Advantage, Clontech) for 10 cycles. Subsequently, proOmpANcoIfw and OmpAHindIIIrv were added and the fragments were amplified for another 20 cycles, and either cloned into pGEM-T (Promega), sequenced and then transferred to pTHV037, or cloned directly into pTHV037 using *Nco*I and *Hind*III sites.

The 3xFLAG insertion in loop 3, and the 2xmyc insertions were created in the same way making use of the following primers: 2xmycOmpAL2RV (5-GAGTTTCTGCTCCAGATCCTCTTCAGAGATGAGTTTCTGCTCACCGTTTTCAACGCTGCCTTTGTAC-3), 2xmycOmpAL2FW (5-GAAGAGGATCTGGAGCAGAAA CTCATCTCTGAAGAGGATCTGGCATACAAAGCTCAGGGCGTTCAAC-3), 2xmycOmpAL3RV (5-GAGTTTCTGCTCCAGATCCTCTTCAGAGATGAGTTTCTG CTCGTTGGATTTAGTGTCTGCACGCC-3), 2xmycOmpAL3FW (5-GAAGAGGAT CTGGAGCAGAAACTCATCTCTGAAGAGGATCTGGTTTATGGTAAAAACCACGACACCG-3), 3xflagOmpAL3RV (5-GTAATCGATGTCATGATCTTTATAAT CACCGTCATGGTCTTTGTAGTCGTTGGATTTAGTGTCTGCACGCC-3), and 3xflagOmpAL3FW (5-GATTATAAAGATCATGACATCGATTACAAGGATGACG ATGACAAGGTTTATGGTAAAAACCACGACACCG-3).

pGI9 was created as follows. OmpA-177 was amplified from pMD005 using the primers proOmpANcoIFW and OmpAAgeIHindIIIRV (5- TCTATAAAGCTTAT ACCGGTGCTGCTTCGCCCTGACC-3), the PCR product digested by *Nco*I and *Hind*III, and was ligated into *Nco*I*/Hind*III digested pGV4. This resulted in pGI8 that contains a silent mutation introducing the *Age*I site in Pro177, for C-terminal addition of the OmpA periplasmic domain DNA fragment. This fragment was obtained by PCR on the LMC500 chromosome, with primers OaperiAgeIFW (5- CTTAAACCGGTAGTT GCTCCGGCTCCAG-3) and OaperiEcoRIHindIIIRV (5- AGATTAAAGCTTAG AATTCAGCGGGGGGATCCTCAAGAGCCTGCGGCTGAGTTACAAC-3) introducing the *Age*I upstream of the periplasmic domain and adding the linker sequence LEDPPAEF downstream. The PCR product was digested with *Age*I*/Hind*III and ligated into *Age*I*/Hind*III digested pGI8. pGI6 was created along similar lines: OmpA-177 containing 3xFLAG in loop 3 was amplified from pGV4 using the primers proOmpANcoIFW and OmpAAgeIHindIIIRV, the PCR product was digested by *Nco*I and *Hind*III, and ligated into *Nco*I*/Hind*III digested pGV4. This resulted in pGI5. Ligating in the *Age*I*/Hind*III periplasmic domain PCR product, used also for pGI9, created pGI6. pGV28 was created by PCR on pB33OA14-SA1 [3] using primers proOmpANcoIFW and OmpAXhoIPstIRV (5- ATTACTGCAGTTAGCTCGAGGGAGCTGCTTCGCCCTG-3), digestion by *Nco*I*/Xho*I, and ligated into *Nco*I*/Xho*I digested pGV14 (unpublished), to get OmpA-177-SS containing SA-1 in loop 1. pGV32 was created by ligation of *Sph*I digested, gel-purified fragments of pGV2 and pGI9, introducing the 3xFLAG loop 2 insertion into the full-length OmpA. pGV33 was created by ligation of *Sph*I digested, gel-purified fragments of pGV28 and pGI9, introducing the SA-1 loop 1 insertion into the full-length OmpA.

### Periplasmic fractionation

TY medium (35 ml) containing 100 μg/ml ampicillin was inoculated with fresh *E. coli* overnight cultures harboring the appropriate plasmids. Cultures were incubated at 37°C until an OD600 of 0.1 was reached. Then IPTG was added to a final concentration of 0.3 mM and the incubation was continued until an OD600 of 1.0 was reached. 25 ml cell culture was pelleted by centrifugation at 4°C (4000 rpm, 15 min), and the cells were fractionated with an osmotic shock according to the procedure described in [4]. The cell pellet was carefully resuspended in 1 ml spheroplast buffer (200 mM Tris-HCl pH 8.0, 0.5 mM EDTA, 0.5 M sucrose, Roche protease inhibitor cocktail) at 4°C. 0.5 ml was stored at -20°C as the whole cell fraction. The remaining 0.5 ml was gently mixed, incubated for 30 minutes on ice and centrifuged in a cooled bench top centrifuge for 10 minutes at 7000 rpm at 4°C. The pellet was resuspended in 0.5 ml of 0.5 mM ice-cold MgCl2, and incubated on ice for another 30 minutes and centrifuged as above. Both supernatant fractions were pooled as the soluble periplasmic fraction (total volume 1 ml), whereas the pellet was dissolved in 1 ml PBS (insoluble fraction). Samples were frozen at -20°C. For western blotting, 5 μl of each sample was mixed with 5 μl sample buffer, boiled and loaded on a 15% SDS-PAGE gel.

1. Amann E, Ochs B, Abel KJ (1988) Tightly regulated tac promoter vectors useful for the expression of unfused and fused proteins in Escherichia coli. Gene 69: 301-315.

2. Den Blaauwen T, Aarsman ME, Vischer NO, Nanninga N (2003) Penicillin-binding protein PBP2 of Escherichia coli localizes preferentially in the lateral wall and at mid-cell in comparison with the old cell pole. Mol Microbiol 47: 539-547.

3. Bessette PH, Rice JJ, Daugherty PS (2004) Rapid isolation of high-affinity protein binding peptides using bacterial display. Protein Eng Des Sel 17: 731-739.

4. Langedijk AC, Spinelli S, Anguille C, Hermans P, Nederlof J, et al. (1999) Insight into odorant perception: the crystal structure and binding characteristics of antibody fragments directed against the musk odorant traseolide. J Mol Biol 292: 855-869.
